# Medication-Related Problems and Interventions Identified and Addressed by Pharmacists Conducting Enhanced Medication Therapy Management Services

**DOI:** 10.3390/pharmacy10050111

**Published:** 2022-09-04

**Authors:** Laura E. Knockel, Yury Kim, Kelly Kent, William R. Doucette

**Affiliations:** 1 College of Pharmacy, University of Iowa, 180 S Grand Avenue, Iowa City, IA 52242, USA; 2 Towncrest Pharmacy, 2306 Muscatine Avenue, Iowa City, IA 52240, USA

**Keywords:** medication-related problems, community pharmacy, continuous medication monitoring

## Abstract

Pharmacists identify, resolve, and document medication-related problems (MRPs) in community pharmacies. Enhanced medication therapy management (eMTM) targets specific situations, such as high-risk medications, while continuous medication monitoring (CoMM) occurs for every patient and is integrated into the dispensing process. This study describes types and frequencies of MRPs and interventions for health plan-directed eMTM and pharmacist-identified CoMM for a cohort of Medicare Part D patients. Pharmacy dispensing and clinical records from one independent community pharmacy in the Midwest were reviewed for patients eligible for eMTM in 2019. Data were coded for medication-related problems and interventions; descriptive statistics were calculated. Forty-seven patients were included in the study, resulting in 439 health plan-directed and 775 pharmacist-identified MRPs and corresponding interventions for a total of 1214 over 12 months. The average age of the patients was 77; they received an average of about 14 medications dispensed over 25 dates. Nonadherence was the most common MRP overall, as well as for the two categories separately. Patient Counseling and Lab Values Needed MRPs were found more often by pharmacists. Continue to Monitor was the most common intervention flagged overall. Medication Discontinued was found more often in health plan-directed interventions; Patient Counseling occurred more frequently in pharmacist-identified interventions. Using pharmacists to identify MRPs can complement health plan-driven eMTM, which can provide more complete medication management. Future work is needed to determine if this approach is reproducible in other pharmacies.

## 1. Introduction

Medication management services (MMSs) include a spectrum of patient-centered, collaborative services that focus on medication appropriateness, effectiveness, safety, and adherence with the goal of improving health outcomes [1]. The term was first used broadly in the United States in 2006 when Medicare Part D, then a new drug benefit plan under the Medicare umbrella, was implemented to assist patients in managing their medications covered under the program. MMSs can include medication therapy management (MTM), comprehensive medication management, or collaborative medication management. The goal of MMSs is to complement already structured patient care plans and practices to help ensure increased safety and efficacy of drug therapy [2]. Pharmacists often play a critical role in executing MMSs, which tend to be directed by insurance plans, in managing patients with specific demographics or health problems. Pharmacists generally also have the most up-to-date information regarding the patient’s medication situation, as the presence of data lags are observed with health plan-directed interactions. Without the data lag, pharmacists can more quickly identify patients with complex medication regimens, who may benefit from close monitoring.

Traditional MTM in Medicare Part D plans includes a uniform set of services provided to all targeted patients. In January 2017, the Centers for Medicare & Medicaid Services launched a five-year Part D Enhanced Medication Therapy Management (eMTM) model [3]. The eMTM model provides payment incentives and regulatory flexibility, allowing Part D drug plan sponsors to determine what types of services they provide and to whom they provide them, with the aim to improve therapeutic outcomes and reduce net Medicare spending. In 2016, only 7.9% of beneficiaries met the required criteria for traditional MTM. For the first 18 months of the eMTM program (January 2017–June 2018), 73.5% of all enrollees were eligible for these patient care services [3].

One of the platform vendors that drug plan sponsors may choose to manage their eMTM patients stratifies patient data to target those most likely to benefit from a Medication Safety Review (MSR) and uses pharmacological characteristics of medications to help reduce a patient’s risk of an adverse drug event by looking at the number of prescribed medications, sedative burden, cognitive impairment, competitive inhibition, and heart rhythm impairment [4,5].

An independent pharmacy in Iowa City, Iowa participates in the described eMTM program and provides services to patients identified by Medicare Part D participating plans. Another form of MMSs provided at this pharmacy for every patient, regardless of their insurance coverage, is Continuous Medication Monitoring (CoMM) [6]. CoMM is integrated into the dispensing process and consists of pharmacists actively monitoring each medication being dispensed, looking to help prevent, identify, and resolve medication-related problems (MRPs). A previous study determined CoMM found an average of 3.4 MRPs per patient over a one-year period [6]. CoMM has also been shown to improve patient adherence [7]. This study’s objective was to describe types and frequencies of MRPs and interventions for health plan-directed eMTM and pharmacist-identified CoMM for a cohort of Medicare Part D patients.

## 2. Materials and Methods

The independent community pharmacy site utilized in this study dispenses approximately 1100 prescriptions per week. This pharmacy provides multiple services including medication synchronization, compounding, immunizations, MTM, and durable medical equipment. Since the start of the COVID-19 pandemic, the pharmacy expanded services to provide COVID-19 testing, COVID-19 vaccinations, and monoclonal antibody treatment. Every patient receives CoMM, a process that has previously been described [6]. All patient interventions are documented in the pharmacy’s proprietary clinical documentation software.

A retrospective analysis of the pharmacist-provided reviews and interventions in Medicare Part D patients eligible for one platform vendor’s eMTM program (MSR) in calendar year 2019 was completed. Patients were excluded if they lived in a nursing home, did not complete an MSR, or were removed from the eMTM program prior to intervention completion. Data were extracted from the pharmacy’s clinical documentation system. The data included a unique patient identification number, age, gender, the number of prescribers, the number of different medications dispensed, the number of dosage forms being taken, the number of high-risk medications, and the dates of medications being dispensed. In addition, for each documented patient encounter (MRP and intervention), data included a unique patient identification number, intervention date, medication name, medication generic product identifier, and a description of the MRP and intervention. Each MRP was accompanied by one intervention. Patients’ medication regimens were classified as low, medium, or high complexity by calculating their Iowa Medication Complexity Score (IMECS) [8]. An IMECS is calculated for a patient using the number of prescribers, the number of different medications dispensed, the number of dosage forms being taken, the number of high-risk medications being taken, and the number of dates medications were dispensed for a patient. This score can be used to prioritize patients who may need more intense medication monitoring due to the complexity of their medication regimen [8].

MRPs and interventions were coded by two researchers, initially using a coding system previously developed at this pharmacy [8]. Coding variability was minimized through two investigators (L.E.K. and Y.K.) refining a coding system. Each investigator coded a subset of cases and then reconvened to discuss and reconcile any differences identified in the coding. The remainder of the coding was completed by one coder (Y.K.), with another (L.E.K.) checking a random set for verification. Any unclear intervention activity was discussed with a third investigator (K.K.) to allow for proper coding. All interventions taking place on the same day as a MSR were classified as health plan-directed; interventions occurring on nonMSR dates were classified as pharmacist-identified. Descriptive statistics (frequencies, means, standard deviations) were calculated for each MRP and intervention using SPSS v. 26 (IBM, Armonk, NY, USA). A glossary of the abbreviations used in this paper is included in Table S1. The University of Iowa Investigational Review Board determined that this study did not meet the definition of human subject research.

## 3. Results

Fifty-seven patients were identified as eligible for an MSR, and 10 patients were excluded for a total of 47 patients in the final analyses. Over half of the patients were female (57.4%, *n* = 27), and the average age (calculated on 31 December 2019) was 77.2 (SD ± 10.5). Patients received a mean of 13.9 medications (SD ± 6.4) and had medications dispensed an average of 25.3 (SD ± 12.6) times over the year. On average, each patient had 5.8 prescribers (SD ± 2.6) writing prescriptions. The mean number of high-risk medications taken was 1.3 (SD ± 1.1). Five patients (10.6%) were considered low complexity; 12 patients (25.5%) were medium complexity, and 30 patients (63.9%) were high complexity, with a range of IMECS from 15–103 [8].

A total of 1214 MRPs were extracted and used for analysis; 36.2% (*n* = 439) were health plan-directed, while 63.8% (*n* = 775) were pharmacist-identified. MRPs are listed in Table 1. Nonadherence was the most common MRP in both health plan-directed (31.7%) and pharmacist-identified (31.9%) cases. Overall, 53.6% (*n* = 207) of the Nonadherence MRPs found were for acute or as needed medications. Therapeutic Duplication was similar across the two groups; High-Risk Medications were 11.6% of MRPs with the health plan-directed group versus 4.3% of the pharmacist-identified.

As each MRP had an intervention, 1214 interventions were extracted and used for analysis (Table 2). Continue to Monitor was the most common intervention in both groups (32.8% of health plan-directed and 37.4% of pharmacist-identified). Discontinuation of a Medication occurred in 20.7% of the health plan-directed interventions, while only 11.1% of the pharmacist-identified interventions were in that category. Patient Counseling occurred in 17.7% of pharmacist-identified interventions versus only 2.3% of health plan-directed interventions.

## 4. Discussion

In this study, pharmacists identified more MRPs and interventions during CoMM than during health plan-directed interactions. The study pharmacy used the health plan-directed MSRs addressing safety as an opportunity to complement the eMTM program, expanding the review to include effectiveness.

Nonadherence was the most common medication-related problem found, which has previously been a well-documented issue [8,9]. This is to be expected as the population studied are older adults on multiple medications, which tends to lower adherence rates [10,11]. Consistent with our findings, Goedken et al. [12] described MRPs identified through CoMM over a 12-month period at the study pharmacy and found Nonadherence to be the most common MRP. That study used a broader patient population, which suggests all patients, not just Medicare patients, can benefit from pharmacists reviewing medication therapy for potential and actual MRPs.

The study pharmacy’s clinical documentation software flagged potential Nonadherence issues during prescription processing, and the pharmacist evaluated the flags. The majority (53.6%) of the medications flagged for a Nonadherence MRP were acute or ‘as needed’ medications, such as influenza vaccinations, colonoscopy preparations, or sublingual nitroglycerin. These MRPs can be used as a conversation starter to discuss efficacy of therapy. For example, an inhaled corticosteroid with a Nonadherence flag can start a discussion with the patient on asthma management and the importance of prophylactic medications. A Nonadherence flag for a shingles vaccination can serve as a reminder for pharmacists to review a patient’s immunization record for other needed vaccinations.

The second most common MRP was Therapeutic Duplication. This may be common for certain disease states, such as hypertension or diabetes, where a patient may need multiple medications to treat one condition. Differentiating between appropriate and inappropriate Therapeutic Duplication requires a pharmacist’s knowledge. For example, a patient may appropriately be on an angiotensin-converting enzyme inhibitor and a thiazide diuretic for hypertension, which would still flag as a Therapeutic Duplication; however, a patient with hypertension who is being prescribed two different angiotensin-converting enzyme inhibitors from two different prescribers would be flagged as an inappropriate Therapeutic Duplication.

The platform vendor identified patients for the eMTM program through risk stratification, based on issues, such as anticholinergic or sedative burden, cytochrome P450 drug interactions, and drug-induced long QT syndrome potential [4,5]. This follows that MRPs, such as high-risk medication and potential fall risk, were more commonly found in the health plan-directed group. Bankes et al. [13] published a study on this eMTM program and found the most common MRPs were Adverse Drug Reaction, Drug Interaction, and Drug Use Without Indication. Unlike this study, MRPs that had Continue, Monitor, Educate, or Referral were excluded as the recommended intervention, which can explain the differences in frequency of MRPs found.

The percentage of total Nonadherence MRPs from health plan-directed was similar to the percentage of pharmacist-identified (31.7% and 31.9%). Medication synchronization programs are another way pharmacists can monitor adherence; these programs have become more common in community pharmacies and have been shown to significantly improve adherence [14,15,16,17]. Patient Counseling Needed occurred more often in the pharmacist-identified group (20.4% to 3.6%). Specifics of the counseling sessions were not captured in the software; it is most likely referring to new medications when patients are most in need of education. Lab Values Needed was more common for pharmacist-identified MRPs as well (10.5% to 3.9%), which illustrates pharmacists looking for both safety and effectiveness of a patient’s medication regimen. Some value-based pharmacy programs use lab values, such as blood pressure, cholesterol, and HbA1c, to measure the impact of services provided, again illustrating the benefits of pharmacist access to lab values [18].

Looking at the documented interventions, 35.7% were Continue to Monitor. As patients age, they tend to have chronic conditions that require more medications and monitoring, which includes evaluating therapy for both effectiveness and safety. These checkpoints help ensure patients are receiving adequate healthcare. Community pharmacists have been traditionally underutilized for patient monitoring, although they are typically the healthcare provider that sees the patient the most [18]. In a study of over 680,000 Medicare beneficiaries, patients visited community pharmacies approximately twice as often as they visited primary care offices [19]. Clinically integrated pharmacy networks have documented the ability of community pharmacists to monitor patients and improve adherence [18,20].

Discontinued Medications were common interventions for both health plan-directed and pharmacist-identified MRPs. This is an important step as pharmacies do not always get notified when a prescriber discontinues a patient’s medication, which can lead to multiple MRPs, such as Therapeutic Duplication or Drug–Drug Interaction. Polypharmacy is a major issue in this patient population, as Medicare Part D patients taking 11 or more medications are almost two times as likely to experience an MRP compared with those taking fewer medications [21]. A systematic review of deprescribing by community-based pharmacists showed that community pharmacists can lead deprescribing interventions and are able to provide monitoring and follow-up for patients to help ensure positive health outcomes [22].

Lab Data Requested/Assessed was the third most common pharmacist-identified intervention. When pharmacists have access to more patient information, such as lab values, they can more easily identify medication-related problems [23]. The frequency with which this intervention was found may be explained through the pharmacy’s participation in a value-based pharmacy program, where there is a focus on obtaining and interpreting a patient’s lab data [24]. Although these patients were not in that program, pharmacists may have become comfortable with asking for lab data and started asking for similar information for other patients. Many disease states, such as diabetes, have lab values that show the effectiveness of the prescribed therapy, while other lab values, such as fasting blood glucose, can be used to monitor safety of therapy. Having this information could improve the ability of the pharmacist to monitor for safe and effective therapy [23], as well as increase efficiency in providing care [25].

The types of MRPs and interventions will vary based on practice setting and characteristics of the cohort of patients. A retrospective chart review of long-term care pharmacy patients found 64% of MRPs were Untreated Indication, and 77% of accepted interventions were to Initiate New Therapy [26]. Pharmacists’ most common MRP and intervention in a surgery ward were Dose Too High or Low and Discontinuing Medications, respectively [27].

This study has several limitations. The clinical documentation system was not designed for research, so there may have been data that were wanted but not collected, such as how many recommendations were accepted by the prescriber. Therefore, we were unable to assess if the documented interventions were accepted or declined by the prescriber. Previous studies have looked at the prescriber acceptance rate of pharmacists’ recommendations. At the study pharmacy, two years of data regarding Iowa Medicaid patients with four or more medications for chronic conditions was reviewed and found physicians accepted 47.4% of pharmacist recommendations; the most common recommendations accepted were to stop or change a medication [28]. Doellner et al. [29] studied the number of pharmacist recommendations accepted by prescribers at a regional grocery store chain pharmacy in Michigan and found that prescribers accepted around 35% of pharmacist recommendations.

These results are based on one pharmacy, which has the workflow, documentation system, and overall buy-in to provide the described level of patient care. Multiple pharmacists were involved in patient care, which may lead to inconsistencies in how MRPs and interventions were documented in the pharmacy, leading to discrepancies in coding, although this does emulate real-world practice. We chose to categorize MRPs using the method previously described by Doucette et al. [8] as it has previously been used when categorizing this pharmacy’s data.

Future directions should include reproducing the combination of eMTM and CoMM in other pharmacies. A focus on upgrading documentation systems or rearranging workflow may be needed to replicate results. Using a standardized method of documenting MRPs may allow for multiple pharmacies’ data to be analyzed and provide insight into the pharmacist’s role in patient care [30]. Research should also continue to explore the prescriber acceptance rates and the factors that impact the acceptance rates.

## 5. Conclusions

Health plans and pharmacists both identified Nonadherence as the most common MRP and Continue to Monitor as the most common intervention. Health plan-identified MRPs and interventions more commonly identified issues with safety of medications, while pharmacist-directed MRPs and interventions dealt more with patient counseling and medication monitoring via lab data. While health plans and pharmacists both identified Nonadherence as the most common MRP, they differed in the most common intervention. Using both insurance plans and pharmacists to identify MRPs increases the number of MRPs identified and resolved, which can provide more complete medication management. This approach can be especially important for older adults taking multiple medications.

## Figures and Tables

**Table 1:** Medication-Related Problems Identified.

Medication-Related Problems	Health Plan- Directed (%)	Pharmacist- Identified (%)	All (%)
Nonadherence	139 (31.7)	247 (31.9)	386 (31.8)
Therapeutic Duplication	64 (14.6)	117 (15.1)	181 (14.9)
Patient Counseling Needed	16 (3.6)	158 (20.4)	174 (14.3)
Meets CMR/eMTM Criteria	103 (23.5)	35 (4.5)	138 (11.4)
Lab Data Needed	17 (3.9)	81 (10.5)	98 (8.1)
High-Risk Medication	51 (11.6)	33 (4.3)	84 (6.9)
Needs Additional Therapy	10 (2.3)	37 (4.8)	47 (3.9)
Potential Fall Risk	16 (3.6)	6 (0.8)	22 (1.8)
Drug–Drug Interaction	8 (1.8)	13 (1.7)	21 (1.7)
Other Issue	1 (0.2)	19 (2.5)	20 (1.6)
Patient Info Request/Report	0 (0.0)	19 (2.5)	19 (1.6)
Other MMS Programs	9 (2.1)	5 (0.6)	14 (1.2)
Medication Allergy Identified/Reported	5 (1.1)	5 (0.6)	10 (0.8)
Total	439	775	1214

CMR = comprehensive medication review; eMTM = enhanced medication therapy management; MMSs = medication management services.

**Table 2:** Interventions Documented.

Interventions	Health Plan- Directed (%)	Pharmacist- Identified (%)	All (%)
Continue to Monitor	144 (32.8)	290 (37.4)	434 (35.7)
Medication Discontinued	91 (20.7)	86 (11.1)	177 (14.6)
Patient Counseling	10 (2.3)	137 (17.7)	147 (12.1)
Lab Data Requested/Assessed	17 (3.9)	88 (11.4)	105 (8.6)
Conducted eMTM Service (MSR)	94 (21.4)	0 (0.0)	94 (7.7)
No Change in Therapy	48 (10.9)	41 (5.3)	89 (7.3)
No Vaccination Needed/Given	10 (2.3)	22 (2.8)	32 (2.6)
Dose Adjusted	9 (2.1)	19 (2.5)	28 (2.3)
Other Action	2 (0.5)	22 (2.8)	24 (2.0)
NonMSR MTM	1 (0.2)	21 (2.7)	22 (1.8)
CMR	7 (1.6)	14 (1.8)	21 (1.7)
Administered Vaccination	0 (0.0)	11 (1.4)	11 (0.9)
Participated in Another Research Study	6 (1.4)	4 (0.5)	10 (0.8)
Current Medication List Created/Reviewed/Provided	0 (0.0)	9 (1.2)	9 (0.7)
Address Patient Requested/Reported	0 (0.0)	4 (0.5)	4 (0.3)
Assessed/Managed Medication Allergy	0 (0.0)	3 (0.4)	3 (0.2)
High-Risk Medication Assessment	0 (0.0)	2 (0.3)	2 (0.2)
Discussed Additional Therapy with Patient/Provider	0 (0.0)	2 (0.3)	2 (0.2)
Total	439	775	1214

eMTM = enhanced medication therapy management; MSR = medication safety review; MTM = medication therapy management; CMR = comprehensive medication review.

## Data Availability

The data presented in this study are available on request from the corresponding author.

## Supplementary Materials

The following supporting information can be downloaded at: https://www.mdpi.com/article/10.3390/pharmacy10050111/s1, Table S1: Glossary of Abbreviations.
