# Supplementary material for: Medication-Related Problems and Interventions Identified and Addressed by Pharmacists Conducting Enhanced Medication Therapy Management Services
Source: Pharmacy (Basel). 2022 Sep 4;10(5):111. doi: 10.3390/pharmacy10050111 (PMC9498483; doi:10.3390/pharmacy10050111)
Supplement: Supplementary file 1 [file pharmacy-10-00111-s001.zip › pharmacy-1873635-supplementary.pdf]

Table S1. Glossary of abbreviations

Abbreviation	Definition
CoMM	Continuous medication monitoring
eMTM	Enhanced medication therapy management
IMECS	Iowa Medication Complexity Score
MMSs	Medication management services
MRP	Medication-related problem
MSR	Medication safety review
MTM	Medication therapy management
